# Electrochemically Controlled Release from a Thin Hydrogel Layer

**DOI:** 10.1021/acsami.3c11786

**Published:** 2023-10-16

**Authors:** Paulina Gwardys, Kamil Marcisz, Damian Jagleniec, Jan Romanski, Marcin Karbarz

**Affiliations:** † Faculty of Chemistry, University of Warsaw, 1 Pasteura, WarsawPL 02-093, Poland; ‡ Biological and Chemical Research Center, University of Warsaw, 101 Żwirki i Wigury Av., WarsawPL 02-089, Poland

**Keywords:** electrochemically controlled releasing system, thermosensitive thin hydrogel layer, *N*-isopropylacrylamide, β-cyclodextrins, inclusion complex

## Abstract

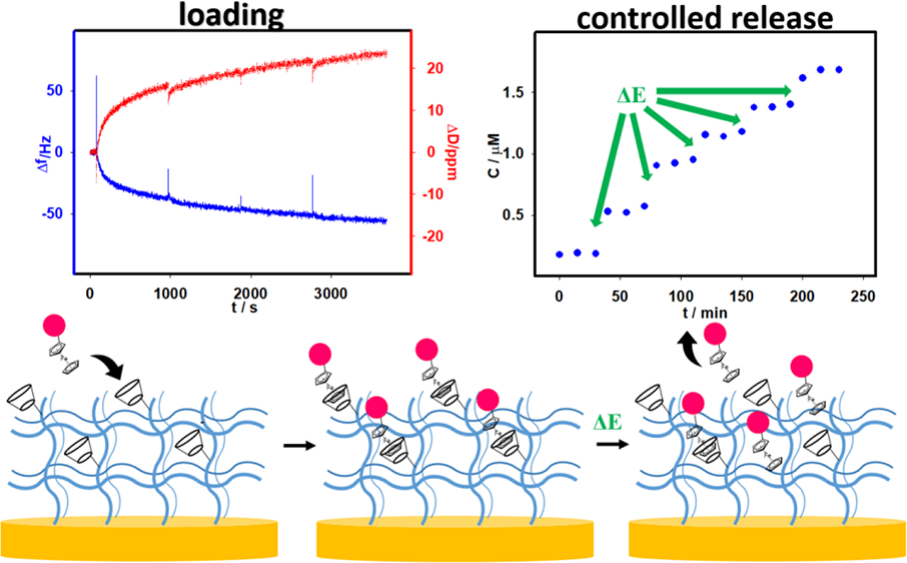

In this study, we present a thermoresponsive thin hydrogel layer based on poly(*N*-isopropylacrylamide), functionalized with β-cyclodextrin groups (p(NIPA-βCD)), as a novel electrochemically controlled release system. This thin hydrogel layer was synthesized and simultaneously attached to the surface of a Au quartz crystal microbalance (QCM) electrode using electrochemically induced free radical polymerization. The process was induced and monitored using cyclic voltammetry and a quartz crystal microbalance with dissipation monitoring (QCM-D), respectively. The properties of the thin layer were investigated by using QCM-D and scanning electron microscopy (SEM). The incorporation of β-cyclodextrin moieties within the polymer network allowed rhodamine B dye modified with ferrocene (RdFc), serving as a model metallodrug, to accumulate in the p(NIPA-βCD) layer through host–guest inclusion complex formation. The redox properties of the electroactive p(NIPA-βCD/RdFc) layer and the dissociation of the host–guest complex triggered by changes in the oxidation state of the ferrocene groups were investigated. It was found that oxidation of the ferrocene moieties led to the release of RdFc. It was crucial to achieve precise control over the release of RdFc by applying the appropriate electrochemical signal, specifically, by applying the appropriate potential to the electrode. Importantly, the electrochemically controlled RdFc release process was performed at a temperature similar to that of the human body and monitored using a spectrofluorimetric technique. The presented system appears to be particularly suitable for transdermal delivery and delivery from intrabody implants.

## 1 Introduction

Hydrogels are a unique class of soft materials composed of cross-linked hydrophilic polymer networks filled with an aqueous solution. These polymer networks can be formed from a variety of polymers cross-linked by covalent or noncovalent interactions. Due to their structure, hydrogels exhibit properties that are characteristic of both solids and liquids. The presence of the polymer network within the hydrogel immobilizes the solvent, resulting in a loss of fluidity. This property allows hydrogels to maintain their shape on a macroscopic scale, while on a microscopic scale, diffusional processes of small molecules/ions can occur within the hydrogel. The unique properties of polymeric hydrogels make them very interesting materials and well-matched to current trends in material research. In addition to hydrogels’ typical properties, such as their absorption of large amounts of water, three-dimensional network that provides specific mechanical properties, thermal and chemical resistance, flexibility, nontoxicity, often biocompatibility, biodegradability, and sorption of heavy metal ions and organic compounds, which are behind the widespread use of gels in many fields, these materials also exhibit other very interesting characteristics. Namely, hydrogels can undergo self-healing, self-assembly, and volume-phase transition (VPT) processes such gels are classified as “smart” materials.^1−4^ In response to particular stimuli, a hydrogel can undergo a significant change in size (*via* the VPT phenomenon), which involves the gel’s conversion from the swollen to the shrunken phase and *vice versa*. These changes originate from a shift in the balance between repulsive intermolecular forces that make the polymer network expand, and attractive forces that make it shrink.^5−8^

These interesting properties of hydrogels have been widely studied and found applications in many areas.^9−13^ The most well-investigated “smart” hydrogel materials include those sensitive to temperature and pH.^14−16^ Sensitivity to those factors makes hydrogels very useful, for instance, in the design of drug delivery systems. In such systems, the hydrogel network not only protects the drug from the hostile environment but also allows it to be released in response to external stimuli.^17−20^ However, the controlled release from hydrogel materials through electrochemical impulses is also highly promising. The combination of these materials with a conductive surface creates the ability to precisely control the release process. From the perspective of applications like transdermal delivery and delivery from intrabody implants, such approaches appear to be particularly intriguing.

Introducing redox-active centers into the polymer network, through covalent bonds, electrostatic interactions, hydrogen bonds, or hydrophobic interactions, may produce an electroactive and electrosensitive gel. The properties of such electrosensitive gels depend strongly on the oxidation state of the redox groups. For instance, a volume-phase transition could occur as a response to a change in the redox probe oxidation state.^21−29^ Introducing ferrocene or benzoquinone moieties to the thermoresponsive p(NIPA) microgels has been found to make the materials electroresponsive. The oxidation state of the redox species has a strong influence on the volume-phase transition temperature. Within a specific temperature range, these microgels can exist in either a swollen or shrunken state, depending on the oxidation state of the electroactive groups.^30,31^ It was found that an electrochemically induced change in the ferrocene moieties’ oxidation state in a microgel with a complex structure allowed the release of a fluorescent dye. This was related with an electrochemical change in hydrophilicity/hydrophobicity, size, or internal structure of the microgel.^32^

Recently, there has been growing interest in coupling polymer gels with cyclic oligosaccharides, particularly cyclodextrins. The modification of gels with cyclodextrins offers promising applications in drug delivery systems and specific sensors.^33,34^ One particularly intriguing property of β-cyclodextrins (βCD) is their ability to form relatively stable inclusion complexes with hydrophobic molecules such as electroactive ferrocene molecules. These host–guest interactions between ferrocene and βCD can be reversibly formed and deformed with a change in a ferrocene oxidation state. This is possible because only the reduced ferrocene form can interact with βCD groups, whereas the oxidized form is much more hydrophilic, and the complex is deformed. Redox stimuli allow for the formation of inclusion complexes between βCD and ferrocene to be controlled. The ability to form reversible host–guest interactions has found an application in a degradable microgel system for doxorubicin release.^35^ Furthermore, hydrogels containing βCD and ferrocene moieties have been used as redox-responsive actuators whose size was altered using redox stimuli.^36^ The formation of inclusion complexes has proved valuable in the development of novel self-healing materials.^37,38^

Anchoring very thin gel layers on the surface of an electrode could increase the possible applications of both materials. Modification of electrode surfaces with environmentally sensitive hydrogel layers could lead to the development of devices such as sensors or biosensors, logic gates, switchable electrochemical systems, electrochemical actuators, or electrochemical valves.^39−45^ In addition, hydrogel and polymer layers can be used as potentially advanced drug delivery and proteins systems.^46^ For instance, Xu et al. used a poly(*N*-isopropylacrylamide-*co*-acrylic acid) microgel layer on an electrode surface as an electrochemically controlled drug release system. The model dye molecules were introduced into the polymer network through electrostatic interactions between the negatively charged polymer network and the positively charged dye. The application of an appropriate reduction potential led to water electrolysis and a corresponding decrease in pH near the electrode surface. The protonation of carboxylic groups in the microgel layer caused the weakness of the electrostatic interaction and, as a consequence, the release of dye molecules from the polymer network.^47^ Wang et al. presented electrochemically induced wireless implants for fluorescein release. The system was based on an electrode modified with a conductive, positively charged polypyrrole nanoparticulate film. The electrostatic interactions were used to introduce negatively charged fluorescein into the film. The electrochemical reduction of the polypyrrole layer led to fluorescein release.^48^

In the present study, a thin hydrogel layer based on p(NIPA) modified with βCD moieties on the Au electrode surface by using electrochemically induced free radical polymerization was obtained. The presence of βCD was used to introduce ferrocene-modified dye molecules to the polymer network through host–guest interactions, which caused the appearance of redox properties. A thermosensitive p(NIPA) thin hydrogel layer with βCD groups was employed to obtain an electrochemically controlled system for releasing rhodamine B modified with electroactive ferrocene (RdFc). In this study, RdFc was employed as a model compound. This compound consists of ferrocene, which plays a pivotal role in the electrochemical release mechanism, and rhodamine B, which aids in the detection of very small quantities of released FcRd. It is worth noting that numerous ferrocene derivatives have been investigated as metallodrugs, and they have demonstrated remarkable effectiveness against various diseases not targeted by existing medications or bioactive compounds.^49−51^ Previous studies of electrochemically controlled release systems from gel layers have predominantly focused on the electrostatic interactions between differently charged polymer chains and the molecules to be released. In contrast, the approach presented here utilizes reversible host–guest inclusion complexes to introduce and release electroactive dye molecules from the polymer network. This strategy offers a different mechanism for controlled release, leveraging the specific interactions between host molecules in the gel network and guest molecules to be released.

## 2 Materials and Methods

### 2.1 Materials

β-Cyclodextrin (β-CD), aminoferrocene, *N*-isopropylacrylamide (NIPA), *N*,*N*′-methylenebis(acrylamide) (BIS), rhodamine B, and ammonium persulfate (APS) were purchased from Aldrich. Sodium nitrate (NaNO_3_), hydrochloric acid (HCl), and potassium chloride (KCl) were purchased from POCh. All reagents were used as received, except NIPA, which was purified with recrystallization from a toluene/hexane mixture (3:7, v/v). All solutions were prepared using high-purity water from a Milli-Q Plus/Millipore purification system (conductivity 0.055 μS·cm^–1^). 6-Amino-β-CD was synthesized according to the procedure described earlier.^52^

### 2.2 Synthesis of Modified β-CD

**Figure 1:**
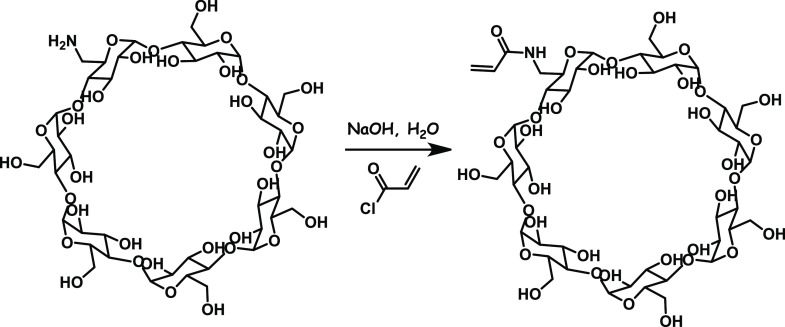
Scheme of the Synthesis of βCD-Am.

In 50 mL of solution of NaHCO_3_ (11.9 M), 6-amino-β-CD (681 mg, 0.6 mmol) was dissolved. Then, the pH of the solution was adjusted to 10 with concentrated solution of NaOH. In the next step, acryloyl chloride (97 μL, 1.2 mmol) was added dropwise at 0 °C. The reaction mixture was stirred for 5 h on ice bath and then left overnight at room temperature. After that, the reaction mixture was concentrated and poured in acetone (700 mL). The white precipitate was filtered off. The crude product was purified by silica gel column chromatography (acetonitrile/water 2:1 v/v, TLC *R*_f_ value: 0.64) to give 6-acrylamido-β-CD (βCD-Am) as a white solid (576 mg, 0.48 mmol, 80% yield, Figure 1).

^1^H NMR (300 MHz, DMSO-*d*_6_) δ: 7.96 (s, 1H), 6.36–6.21 (m, 1H), 6.13–5.98 (m, 1H), 5.94–5.49 (m, 15H), 4.95–4.73 (m, 7H), 4.58–4.36 (m, 6H), 3.85–3.21 (m, overlaps with HOD).

HRMS (ESI): calcd for C_45_H_73_NO_35_Na [M + Na]^+^: 1210.3855, found: 1210.3888.

### 2.3 Synthesis of Ferrocene-Modified Rhodamine B

**Figure 2:**
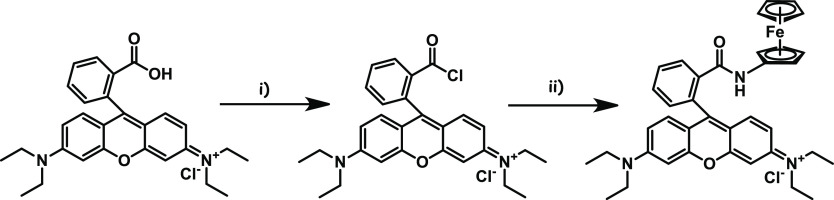
Synthesis of compound ferrocene-modified rhodamine B. Reagents and conditions: (i) oxalyl chloride, dry dichloromethane, dimethylformamide, argon atmosphere, 12 h, rt, quantitative; (ii) aminoferrocene, dry dichloromethane, triethylamine, 24h, rt, 59%.

To the solution of rhodamine B (400 mg, 0.83 mmol) in 10 mL of dry dichloromethane, two drops of dimethylformamide and oxalyl chloride (130 μL, 1.51 mmol) were added in an argon atmosphere. After being stirred for 12 h at room temperature, the reaction mixture was concentrated and the crude rhodamine B chloride obtained was used in the next step without further purification. Then, aminoferrocene (170 mg, 0.84 mmol) was dissolved in dry dichloromethane and added dropwise to the solution of rhodamine B chloride along with 400 μL triethylamine (Figure 2). After stirring overnight at room temperature, the reaction mixture was concentrated, and the residue was purified by silica gel column chromatography (2% methanol in chloroform) (TCL *R*_f_ value: 0.81) to produce the final compound as a red solid (324 mg, 59% yield).

^1^H NMR (300 MHz, DMSO-*d*_6_) δ: 7.87–7.79 (m, 1H), 7.58–7.43 (m, 2H), 6.99–6.89 (m, 1H), 6.55–6.25 (m, 6H), 4.45–4.31 (m, 2H), 3.98–3.81 (m, 6H), 3.45–3.35 (m, overlaps with HOD), 1.21–0.95 (m, 12H).

^13^C NMR (75 MHz, DMSO-*d*_6_) δ: 166.3, 154.7, 152.7, 148.8, 133.4, 129.8, 128.8, 128.7, 123.9, 122.6, 108.4, 106.0, 97.6, 95.0, 69.4, 65.7, 64.5, 60.4, 44.1, 12.9.

HRMS (ESI): calcd for C_38_H_39_FeN_3_O_2_Na [M + Na]^+^: 648.2284, found: 648.2299.

### 2.4 Instrumental Measurements

#### 2.4.1 Electrochemical Measurements

Electrochemical measurements were performed with a CH Instruments 400B potentiostat. The three-electrode system was used. A platinum wire and a saturated silver chloride electrode were used as the counter and reference electrode, respectively. An Au quartz crystal microbalance with a dissipation (QCM-D) electrode was used as a working electrode. All electrodes were kept in a self-modified electrochemical cell from the manufacturer. To reduce the noise, the electrochemical cell was placed in a Faraday cage.

#### 2.4.2 QCM-D Measurements

QCM-D measurements were performed with a QEM 401 (Q-Sense, Biolin Scientific) instrument equipped with 4.95 MHz AT-cut gold-coated quartz crystals. Before the experiments, the electrode surface was cleaned with “hot-piranha” solution for 10 min to remove organic pollutants, then rinsed with water and ethanol, and then dried in a stream of argon gas. Next, the electrode was mounted in the electrochemical cell. Data from QCM-D measurements were used to calculate layer thickness, with Dfind software, with a viscoelastic Voigt-based included model.

#### 2.4.3 Scanning Electron Microscopy (SEM) Measurements

The morphology of the gel samples was analyzed with a Zeiss Merlin field emission scanning electron microscope. Before the examination, one sample of the electrode modified with the thin hydrogel layer was lyophilized, and a second sample was dried in air. Samples before the analysis were coated with a thin, approximately 3 nm layer of Au–Pd alloy using a Polaron SC7620 mini-sputter coater.

#### 2.4.4 Spectrophotometry

Spectroscopy measurements were obtained with a Thermo Spectronic Unicam UV500 spectrophotometer and Hitachi F-7100 fluorescence spectrophotometer in water at 22 °C.

## 3 Results and Discussion

**Figure 3:**
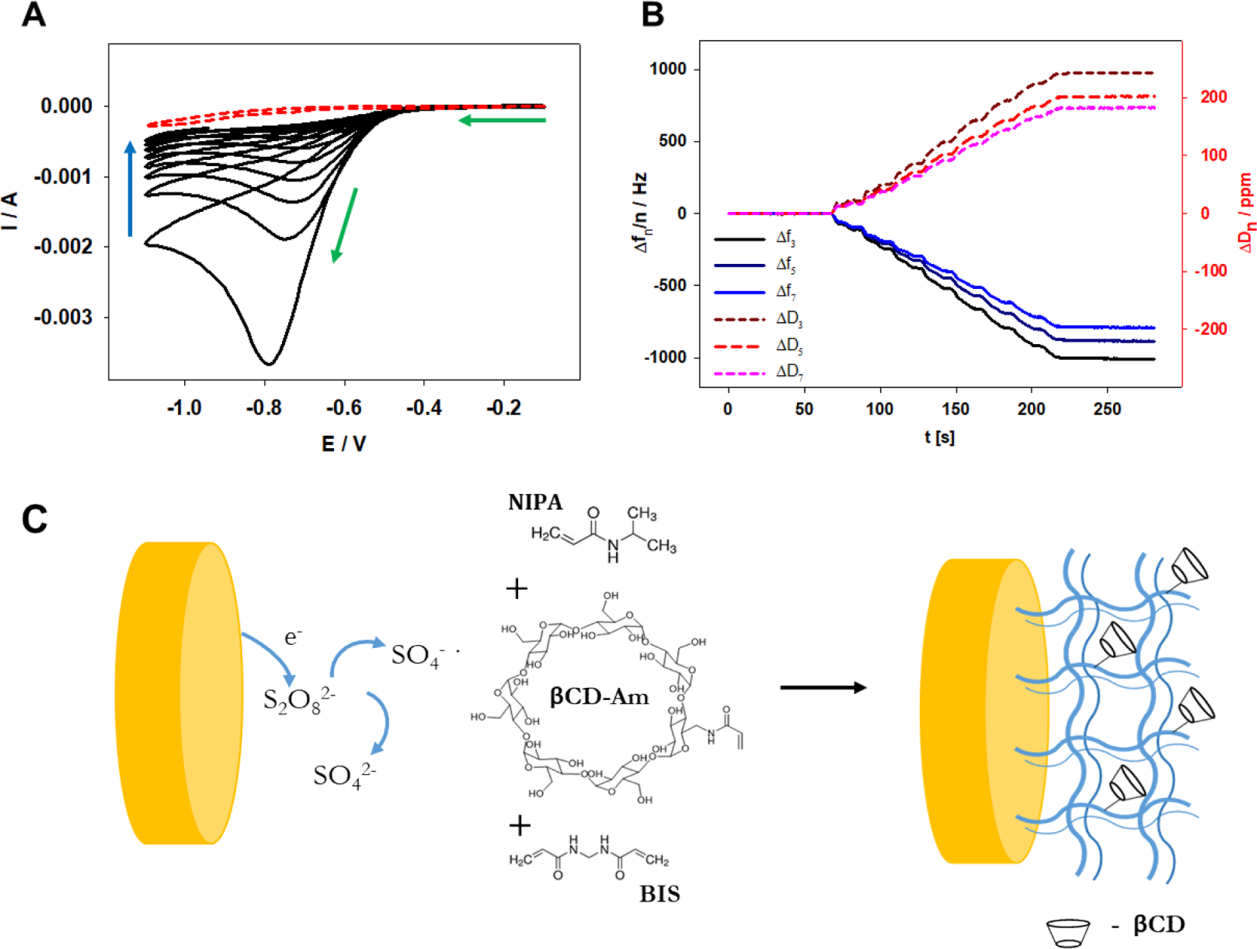
(A) Voltammograms obtained during the p(NIPA-βCD) gel layer deposition on the Au QCM-D electrode (with a geometric area of 0.97 cm^2^) surface with (black lines) and without (red dashed lines) added initiator (the green arrows indicate the scan direction, and the blue arrow indicates the subsequent cyclic curves recorded) and (B) simultaneously registered frequency (Δ*f*) and dissipation (Δ*D*) shifts and curves for the third, fifth, and seventh overtones. Scan rate: 100 mV/s; supporting electrolyte: 0.2 M NaNO_3_, *T* = 20 °C. (C) Scheme of electrode surface modification with the p(NIPA-βCD) gel layer.

A thin p(NIPA-βCD) hydrogel layer was synthesized on the gold electrode surface by using the electrochemically induced free radical polymerization method. For this purpose, NIPA and βCD-Am were used as monomers and BIS as a cross-linking agent. The total concentration was set at 0.5 M (92% NIPA, 7% βCD-Am, and 1% BIS). The βCD-Am, NIPA, and BIS monomers were dissolved in 1.9 mL of supporting electrolyte (0.1 M NaNO_3_). The prepared solution was placed in an electrochemical cell with an Au QCM-D electrode. The monomer solution was degassed with a stream of argon for 15 min, and then, 0.1 mL of 0.2 M APS, as an initiator, was added to the solution. The polymerization process was initiated by applying the appropriate negative potential. The electroinduced polymerization process was carried out with cyclic voltammetry and monitored with the QCM-D technique. The reaction was stopped after registered delta frequency (third overtone) reached approximately 1000 Hz. The obtained cyclic voltammograms and simultaneously obtained quartz crystal frequency and dissipation shifts are presented in Figure 3A,B. The scheme of the polymerization process on the electrode surface is presented in Figure 3C. The obtained p(NIPA-βCD) hydrogel layer was washed with Milli-Q water several times to remove unreacted reagents.

Since the p(NIPA-βCD) hydrogel layer was mostly based on a thermoresponsive polymer, the temperature-dependent properties were examined. For this purpose, the QCM-D technique was used to study the volume-phase transition phenomenon. The mass change on the quartz crystal microbalance electrode surface is described by the Sauerbrey eq 1:
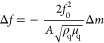
1where Δ*f* is the frequency shift, *f*_0_ is the quartz oscillation frequency in the fundamental mode, *A* is the piezoelectrically active surface area, ρ_q_ is the density of quartz, and μ_q_ is the shear modulus of quartz.^53^ However, the Sauerbrey equation is valid only for thin, rigid, and homogeneous layers. For hydrogel layers with viscoelastic properties, more information is provided by the combination of frequency and energy dissipation results.^54,55^ In this case, the assumptions leading to eq 1, which assumes linearity between Δ*f* and Δ*m*, are violated. Both experimental and modeling approaches have demonstrated that for viscoelastic films in liquid media, eq 1 underestimates the coupled mass.^56^ To determine the thickness of the gel layer, the Voigt model can be applied. In this model, the frequency shift and energy dissipation are dependent on several factors, including the thickness, density, viscosity, and shear modulus of the deposited material, as well as the density and viscosity of the contact fluid. Voinova et al.^57^ have presented the relationship between these parameters and changes in frequency and energy dissipation. By analysis of the recorded Δ*f* and Δ*D* data using the Voigt model, it becomes feasible to derive valuable insights into the characteristics of the adsorbed layer, including its density, thickness, shear viscosity, and elasticity. The number of parameters to be determined by the fit should not exceed the minimum number of independent input data. That is, information from Δ*fn* and Δ*Dn* (*n* = 1, 3, 5...).^58^ However, it is worth noting that there is not a unique solution for viscosity and density, and one of these parameters must either be independently determined or assumed.^59^

**Figure 4:**
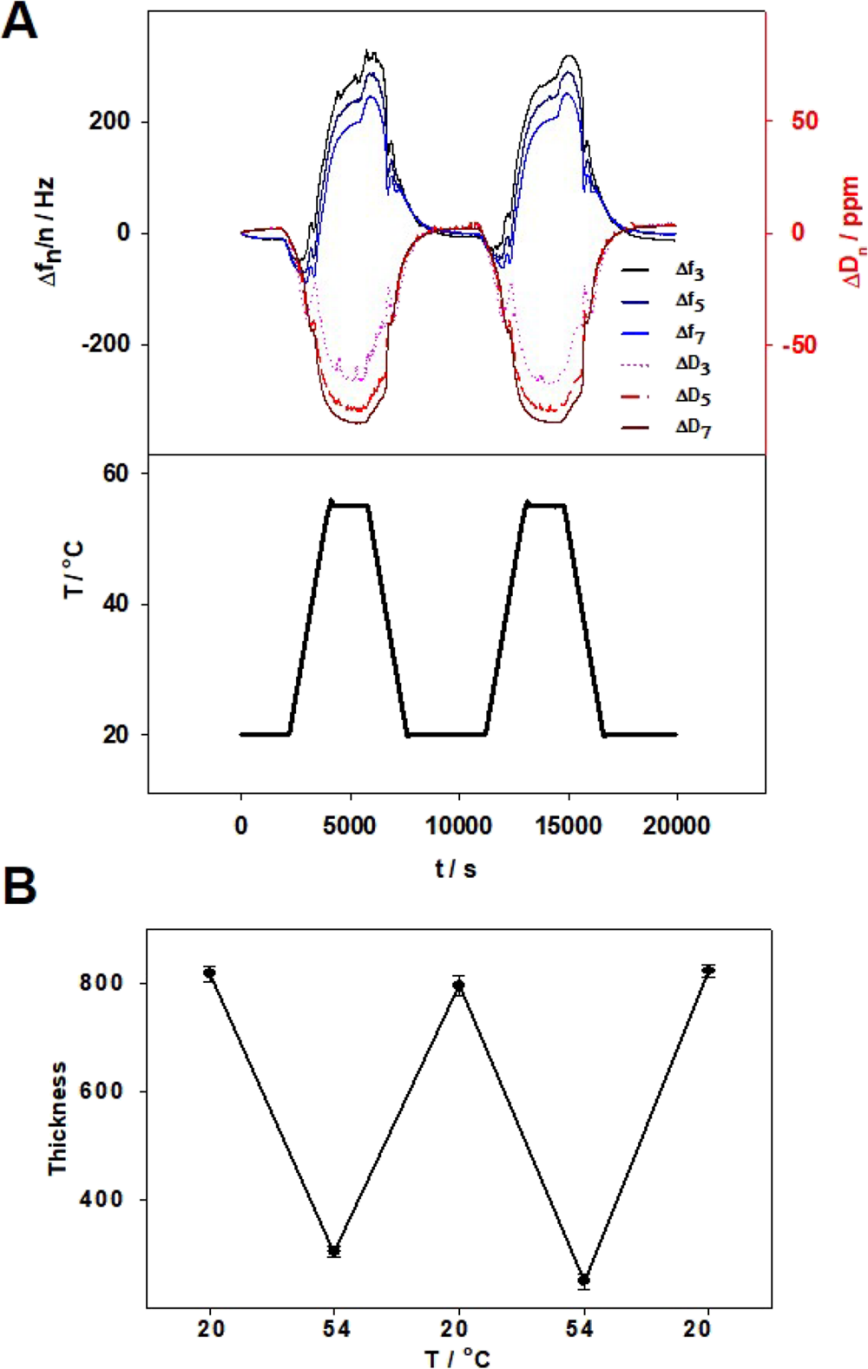
(A) Temperature-dependent frequency and dissipation shifts (curves for third, fifth, and seventh overtones) obtained for the Au QCM-D electrode modified with the p(NIPA-βCD) hydrogel layer. (B) p(NIPA-βCD) hydrogel layer thickness changes, calculated from frequency/dissipation shifts, during the temperature change.

The frequency and dissipation shifts obtained with the p(NIPA-βCD) hydrogel layer-modified Au QCM-D electrode upon the temperature change are presented in Figure 4A. As is evident, a temperature increase caused an increase in registered frequency and decrease in dissipation shift related with the gel layer shrinking process: water was removed from the polymer network and the film became more rigid. On the other hand, a temperature decrease led to the opposite effect: a decrease in frequency and increase in dissipation shifts related with water absorption from the environment; the gel simply becomes much more viscoelastic. The presence of hydrophilic βCD groups in the polymer network caused a shift in the temperature of the volume-phase transition to approximately 36 °C, in comparison to the typical 32 °C for pNIPA gels. The obtained data allows the p(NIPA-βCD) hydrogel layer thickness to be calculated with a viscoelastic Voigt-based model. It was found that the hydrogel layer was approximately 820 nm thick in the swollen state (20 °C) and approximately 200 nm thick in the shrunken state (55 °C) (Figure 4B). The transition from the swollen to shrunken state and *vice versa* was well reproducible and repeatable.

**Figure 5:**
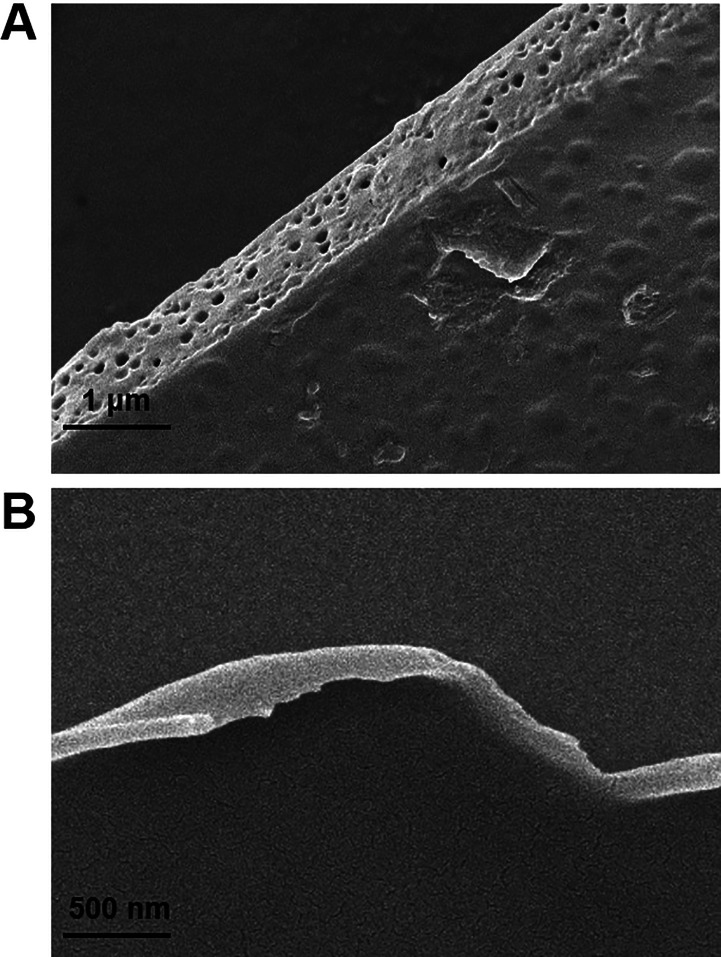
SEM micrographs of (A) lyophilized and (B) dried partially detached p(NIPA-βCD) hydrogel layers on the Au QCM-D electrode surface.

Next, the p(NIPA-βCD) hydrogel layer morphology was examined with an SEM technique. For this purpose, the modified electrode was cut into small pieces, one portion of them was lyophilized, and the other was dried in air. As can be seen in Figure 5A, the detached, lyophilized layer had a porous structure, approximately 750 nm thick. For comparison, the dried sample is shown in Figure 5B, with the determined layer thickness being approximately 150 nm. It can be assumed that the lyophilized gel layers’ thickness should be similar to the swollen state and that of the dried ones similar to the shrunken state. As can be seen, the calculated layer thickness from QCM-D for the swollen and shrunken states is somewhat overestimated compared to the values obtained from SEM microimages.

**Figure 6:**
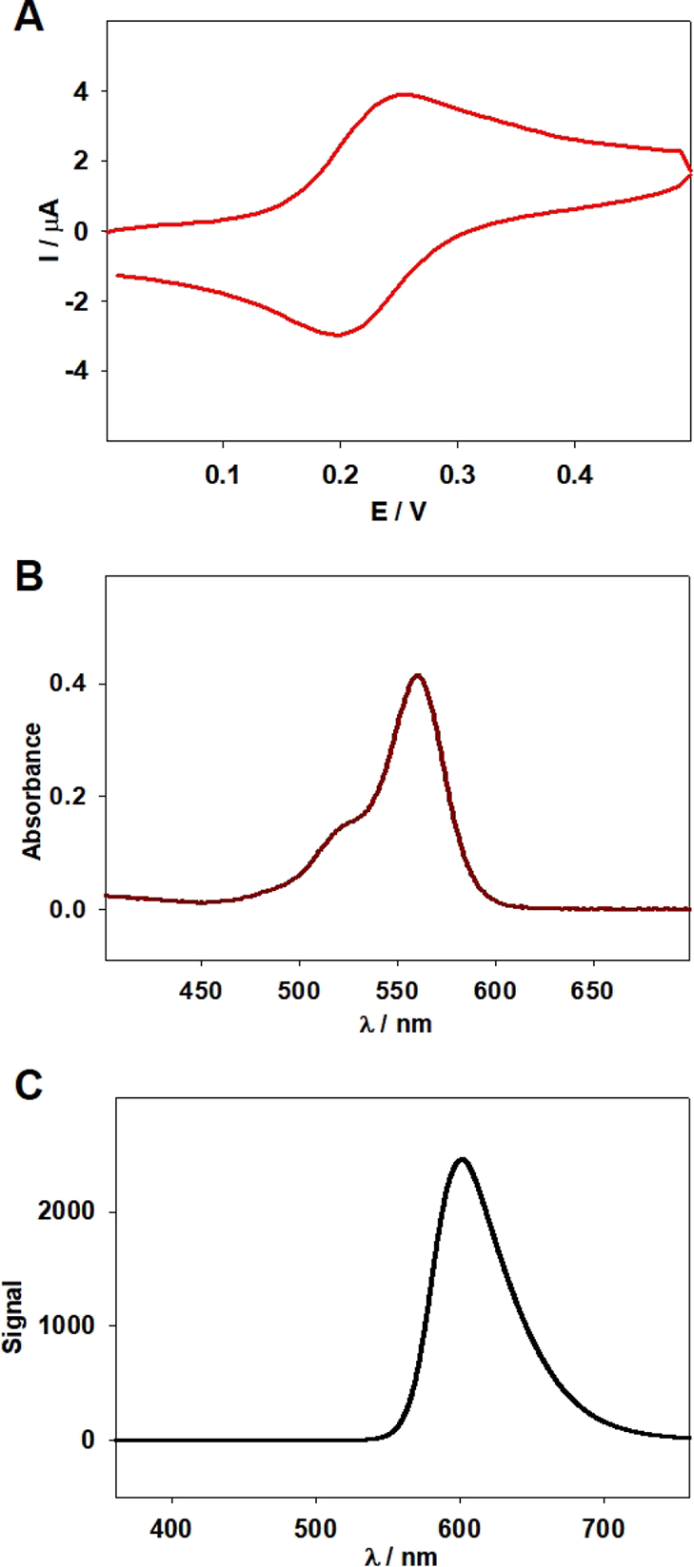
(A) Voltammograms obtained with a 2 mm diameter Au disk electrode in a 1 mM RdFc solution. Supporting electrolyte: 0.1 M KCl, *T* = 20 °C, *v* = 50 mV/s. (B) UV–vis spectra of 1 mM RdFc solution. (C) Fluorescence excitation spectrum of 1 mM RdFc solution, excitation wavelength: 561 nm.

Next, the properties of ferrocene-modified rhodamine B (RdFc) were examined. The electroactive properties were studied with cyclic voltammetry. In Figure 6A, a pair of peaks characteristic for ferrocene moieties can be observed. The shape of the obtained voltammetric responses is typical for a purely infinite diffusional process. The difference between the oxidation and reduction peak potentials was found to be approximately 63 mV. This value is somewhat greater than the theoretical value of 2.22RT/nF (0.056 V for 20 °C) for a one-electron perfectly reversible process. In Figure 6B, the UV–vis spectra of the RdFc solution are presented. The typical peaks for the rhodamine B species are visible at 520 and 561 nm. Consecutively, a spectrofluorimetric spectrum was obtained with 561 nm wavelength excitation. The registered spectra are presented in Figure 6C; the fluorometric response is very well-defined and confirms that ferrocene-modified rhodamine B could be determined with fluorescence spectrophotometry.

**Figure 7:**
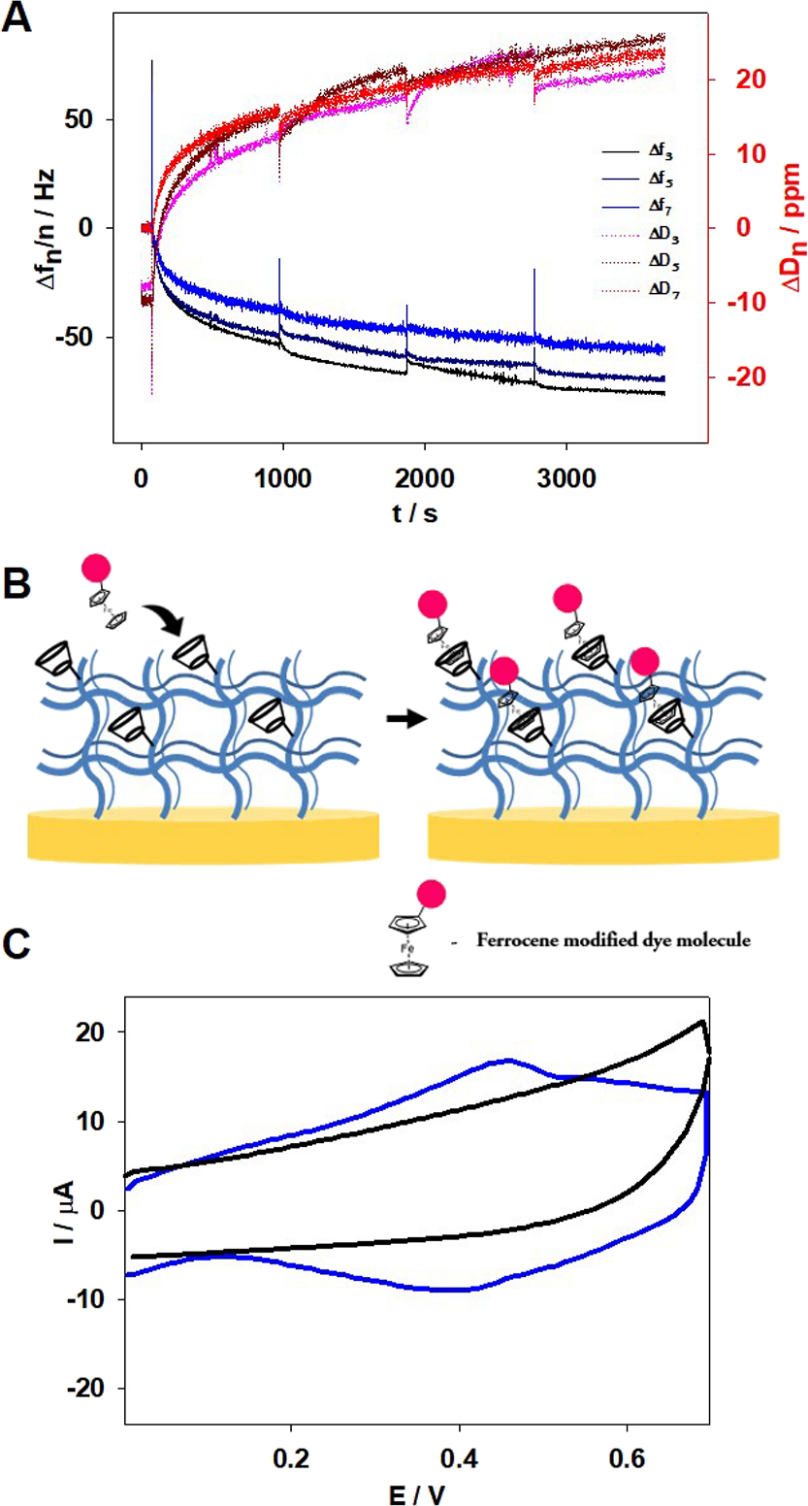
(A) QCM-D frequency (Δ*f*) and dissipation (Δ*D*) shifts obtained during the consecutive additions of RdFc to the p(NIPA-βCD) hydrogel layer on the Au QCM-D electrode surface (curves for third, fifth, and seventh overtones). (B) Scheme of the process of introducing RdFc molecules to the electrode modified with the p(NIPA-βCD) hydrogel layer. (C) Cyclic voltammograms were obtained with the p(NIPA-βCD)-modified electrode with RdFc introduced (blue line) and without it (black line). Supporting electrolyte: 0.1 M KCl, *T* = 20 °C, *v* = 50 mV/s (C).

In the next step, the incorporation of RdFc into the p(NIPA-βCD) hydrogel layer was studied. For this purpose, 50 μL of RdFc (1 mM in 0.1 M HCl) was added every 15 min to the QCM-D electrochemical cell, with the modified electrode mounted and filled with 2 mL of water at 37 °C. The process was monitored with the QCM-D technique. As Figure 7A shows, the first electroactive dye addition caused a decrease in registered frequency to approximately −50 Hz for the third overtone, related with the mass increase on the electrode surface. The formation of an inclusion complex between βCD in the hydrogel layer and ferrocenium in RdFc took place, observed as the increase in mass on the electrode surface. Each successive addition of RdFc solution caused a smaller decrease in registered frequency shifts, and after the fourth addition, the decrease was insignificant. Therefore, after 1 h, the process was stopped. The temperature was set to 20 °C and left for 30 min, to increase the accumulation process through the “sponge”-like absorption in the gel swelling process. Next, the electrode modified with p(NIPA-βCD-RdFc) hydrogel layer was washed several times, to remove unbounded dye molecules, and left overnight in water. The scheme of the introduction of RdFc moieties into the p(NIPA-βCD) hydrogel layer on the electrode surface, through the inclusion complex formation, is shown in Figure 7B.

Subsequently, the electrochemical properties were examined. Figure 7C shows voltammograms obtained by using p(NIPA-βCD) and p(NIPA-βCD-RdFc)-modified electrodes. In the case of the electrode modified with p(NIPA-βCD-RdFc), a thin gel layer pair of peaks can be observed (blue line). The shape of the obtained voltammetric response is not typical either for a pure infinite diffusional or for a pure surface-confined process. Instead, the shape is typical for electrodes modified with electrically nonconductive, polymer redox films.^60^ In addition, the obtained voltammetric response is shifted in a higher potential range compared to the data shown in Figure 6A (RdFc in solution). These findings can be explained by the fact that RdFc compounds were accumulated/immobilized in the thin gel layer by forming inclusion complexes between ferrocene molecules and βCD groups attached to the polymer network. No characteristic signals (black solid lines) were observed for voltammetrograms recorded using a p(NIPA-βCD)-modified electrode without RdFc.

**Figure 8:**
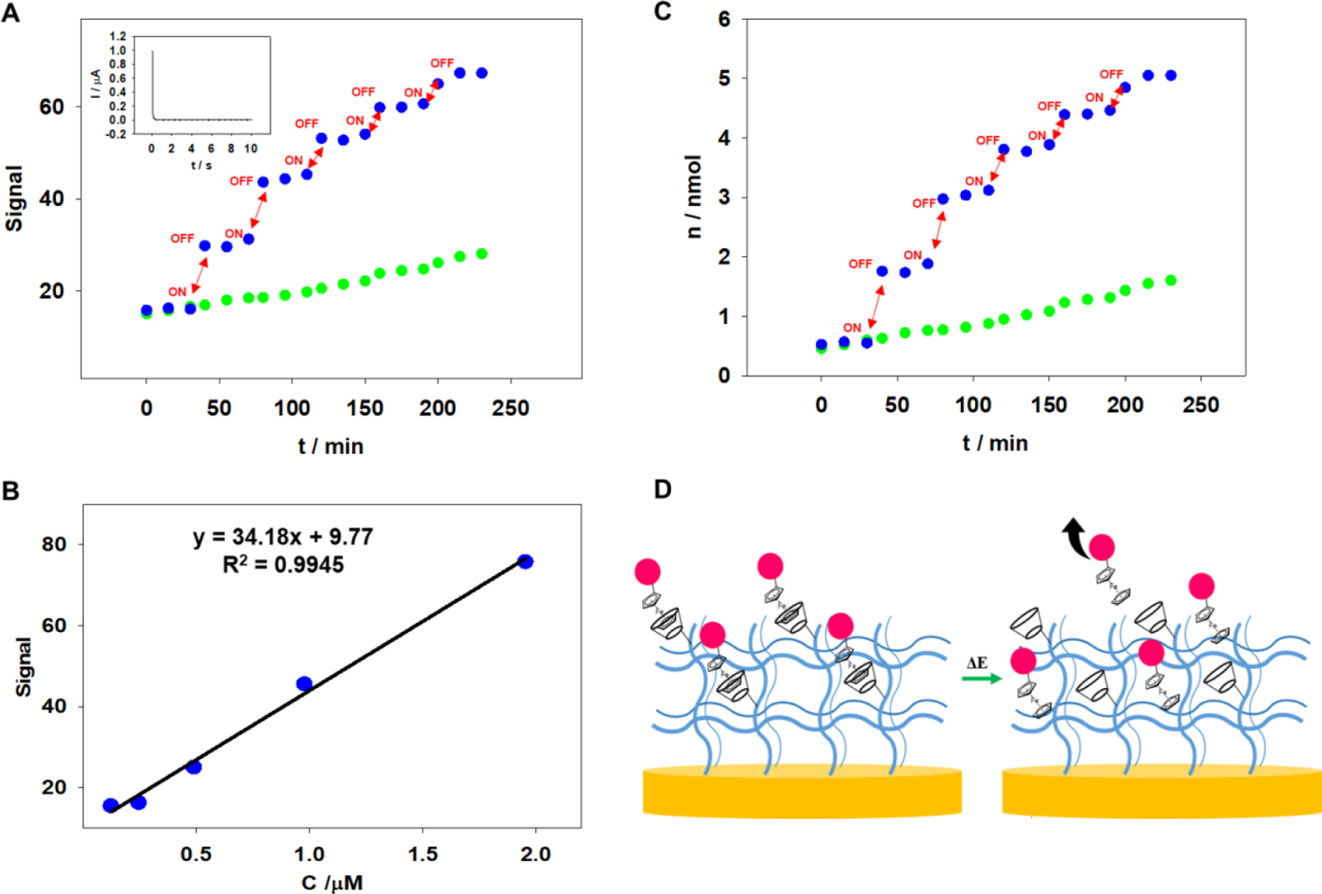
RdFc spectrofluorimetric release profile from the p(NIPA-βCD-RdFc) hydrogel layer on the electrode surface with applied potential (blue dots) and without (red squares). (A) Temperature 37 °C, supporting electrolyte: 0.1 M KCl, arrows indicate electrochemical potential application, 0.5 V for 10 min. (B) RdFc spectrofluorimetric calibration curve. (C) RdFc molecule molar release profile from the p(NIPA-βCD-RdFc) hydrogel layer on the electrode surface with (blue dots) and without applied potential (red squares). (D) Scheme of electrochemical induced RdFc molecules release from the p(NIPA-βCD-RdFc) hydrogel layer on the electrode surface.

The capacity for the reversible formation/deformation of inclusion complexes, depending on the ferrocene oxidation state, between RdFc molecules and βCD groups in the hydrogel polymer network was used as a mechanism for releasing dye moieties from the hydrogel layer. The application of appropriate positive potential to the modified electrode led to the oxidation of RdFc groups; the formation of ferrocene cation should cause the deformation of inclusion complexes, with simultaneous release of RdFc moieties to the environment. To study this process, a p(NIPA-βCD-RdFc)-modified electrode was placed in an electrochemical cell filled with 3 mL of 0.1 M KCl solution as the supporting electrolyte, and the temperature was maintained at 37 °C to mimic human body conditions. Initially, three fluorometric measurements were taken at an excitation wavelength of 561 nm after 0, 15, and 30 min. During each measurement, 1 mL of sample was collected from above the hydrogel layer and returned after analysis. Subsequently, a chronoamperometric technique was employed with a 0.5 V oxidation potential applied to the electrode for 10 min, following which fluorometric measurements were performed again. In this experiment, only oxidation potential was applied to avoid that some of the released/oxidized RdFc compounds could be reduced on the electrode surface and recaptured by the hydrogel layer. This potential application and release monitoring procedure were repeated a total of 5 times, and the results are plotted in Figure 8A. Upon applying potential, a notable increase in the measured signal was observed, indicating the release of RdFc from the hydrogel layer into the solution. Each potential application yielded a similar effect, leading to an increase in the measured fluorometric signal. Following each appropriate potential application to the modified electrode, the registered signal remained quite stable until the subsequent potential step occurred. Additionally, the release of RdFc moieties without applying a potential was also investigated. It was observed that the release process still occurred but the registered signals were significantly smaller than after electrochemical measurements. The amount of released RdFc was also calculated. For this purpose, a calibration curve was constructed using the spectrofluorimetric technique to determine the RdFc concentration, and the results are illustrated in Figure 8B. In Figure 8C, the calculated molar amount of RdFc that was released from the polymer network is presented. The total molar amount of RdFc released from the p(NIPA-βCD-RdFc) hydrogel layer on the electrode surface, after five repetitions of the applied potential, was approximately 5 nmol. This value is approximately 60% of the total amount of RdFc loaded into the gel layer, which was estimated from fluorometric measurements. These findings provide crucial insights into the release mechanism and the effectiveness of the electrochemical process in facilitating the release of RdFc moieties from the hydrogel layer.

## 4 Conclusions

A thermosensitive thin hydrogel layer, based on *N*-isopropylacrylamide cross-linked with *N*,*N*′-methylenebis(acrylamide) and copolymerized with β-cyclodextrin acrylamide derivative, was successfully synthesized on a Au QCM-D electrode surface using the electrochemically induced free radical polymerization method. The layer-attaching process was monitored with a quartz crystal microbalance with an energy dissipation technique. Thermoresponsiveness of the layer was examined using QCM-D, with calculated layer thickness of approximately 820 nm in a swollen state and approximately 200 nm in a shrunken state. The thicknesses of the layer from SEM images were quite similar to those calculated from QCM-D. Due to the presence of βCD groups in the polymer network, ferrocene-modified rhodamine B was successfully introduced to the thin gel layer by forming inclusion complexes. Then, the possibility of reversible host–guest interaction formation between ferrocene and βCD was investigated in terms of a controlled release process of dye moieties from the hydrogel layer. It was demonstrated that application of the appropriate potential to the electrode surface modified with a thin hydrogel layer led to the oxidation of ferrocene groups, with simultaneous complex hydrophobic–hydrophilic balance shift and, as a result, a release of the RdFc from the polymer network. It was crucial to achieve precise control over the release of RdFc by applying the appropriate electrochemical signal. The release process occurred at a temperature near that of the human body, and the amount of substance released from the hydrogel layer could be controlled with an electrochemical signal. The obtained results allow us to conclude that the thermoresponsive p(NIPA-βCD) hydrogel layer can be successfully used as an electrochemically controlled release system. In particular, the mechanism used holds significant promise for the controlled delivery of ferrocene derivatives that are promising metallodrugs.

## Data Availability

Data will be available on request.
